# Quality Outcomes in Appendicitis Care: Identifying Opportunities to Improve Care

**DOI:** 10.3390/life10120358

**Published:** 2020-12-18

**Authors:** Syed Mohammad Umar Kabir, Magda Bucholc, Carol-Ann Walker, Opeyemi O. Sogaolu, Saqib Zeeshan, Michael Sugrue

**Affiliations:** 1 Donegal Clinical Research Academy and Department of Surgery Letterkenny University Hospital, Letterkeny, Co. F92 AE81 Donegal, Ireland; umarkabir@hotmail.com (S.M.U.K.); ope.sogaolu@gmail.com (O.O.S.); Saquib.zeeshan3@hse.ie (S.Z.); 2 Intelligent Systems Research Centre, University of Ulster, Magee Campus, Londonderry BT48 7JL, UK; m.bucholc@ulster.ac.uk; 3 EU INTERREG Emergency Surgery Outcome Advancement Project, Centre for Personalised Medicine, X728 HG Letterkenny, Ireland; Carolann.walker@hse.ie

**Keywords:** appendicitis, appendectomy, re-admission, Alvarado score, Appendicitis Inflammatory Response score, Adult Appendicitis Score, negative appendectomy rate

## Abstract

Introduction: Appendicitis is one of the most common causes of acute abdominal pain requiring surgical intervention, but the variability of diagnosis and management continue to challenge the surgeons. *Aim*: This study assessed patients undergoing appendectomy to identify opportunities to improve diagnostic accuracy and outcomes. Methods: An ethically approved retrospective cohort study was undertaken between March 2016 and March 2017 at a single university hospital of all consecutive adult and paediatric patients undergoing appendectomy. Demographic data including age, gender, co-morbidities, presentation and triage timings along with investigation, imaging and operative data were analysed. Appendicitis was defined as acute based on histology coupled with intraoperative grading with the American Association for the Surgery of Trauma (AAST) grades. Complications using the Clavien–Dindo classification along with 30-day re-admission rates and the negative appendectomy rates (NAR) were recorded and categorised greater and less than 25%. The use of scoring systems was assessed, and retrospective scoring performed to compare the Alvarado, Adult Appendicitis Score (AAS) and the Appendicitis Inflammatory Response (AIR) score. *Results*: A total of 201 patients were studied, 115 male and 86 females, of which 136/201 (67.6%) were adults and 65/201 (32.3%) paediatric. Of the adult group, 83 were male and 53 were female, and of the paediatric group, 32 were male and 33 were female. Median age was 20 years (range: 5 years to 81 years) and no patient below the age of 5 years had an appendectomy during our study period. All patients were admitted via the emergency department and median time from triage to surgical review was 2 h and 38 min, (range: 10 min to 26 h and 10 min). Median time from emergency department review to surgical review, 55 min (range: 5 min to 6 h and 43 min). Median time to operating theatre was 21 h from admission (range: 45 min to 140 h and 30 min). Out of the total patients, 173 (86.1%) underwent laparoscopic approach, 28 (13.9%) had an open approach and 12 (6.9%) of the 173 were converted to open. Acute appendicitis occurred in 166/201 (82.6%). There was no significant association between grade of appendicitis and surgeons’ categorical NAR rate (*p* = 0.07). Imaging was performed in 118/201 (58.7%); abdominal ultrasound (US) in 53 (26.4%), abdominal computed tomography (CT) in 59 (29.2%) and both US and CT in 6 (3%). The best cut-off point was 4 (sensitivity 84.3% and specificity of 65.7%) for AIR score, 9 (sensitivity of 74.7% and specificity of 68.6%) for AAS, and 7 (sensitivity of 77.7% and specificity of 71.4%) for the Alvarado score. Twenty-four (11.9%) were re-admitted, due to pain in 16 (58.3%), collections in 3 (25%), 1 (4.2%) wound abscess, 1 (4.2%) stump appendicitis, 1 (4.2%) small bowel obstruction and 1 (4.2%) fresh rectal bleeding. CT guided drainage was performed in 2 (8.3%). One patient had release of wound collection under general anaesthetic whereas another patient had laparoscopic drain placement. A laparotomy was undertaken in 3 (12.5%) patients with division of adhesions in 1, the appendicular stump removed in 1 and 1 had multiple collections drained. Conclusion: The negative appendectomy and re-admission rates were unacceptably high and need to be reduced. Minimising surgical variance with use of scoring systems and introduction of pathways may be a strategy to reduce NAR. New systems of feedback need to be introduced to improve outcomes.

## 1. Introduction

Acute appendicitis remains one of the most common presentations of acute abdominal pain in both adults and children, with a prevalence of approximately 7% and an incidence of 11 cases/10,000 population per year [1]. History and physical examination, along with laboratory testing, remain the keys to diagnosis, aided with imaging and scoring modalities [2,3,4]. There is increasing recognition of the need to improve decision making in management of acute appendicitis and reduce the negative appendectomy rates which approach 25% [5,6,7]. Integrated scoring systems may reduce unnecessary surgery [4,8,9]. In addition, the identification of complicated appendicitis has become a further goal to avoid delay in surgery and reduce the increase in complications seen in that sub-group [10]. Delay in surgery in patients with complex appendicitis, especially those with perforation and peritonitis, may result in increased post-operative complications [11].

For centuries, acute appendicitis has been managed surgically [12], and a laparoscopic approach is now preferred due to lower complication rates [8,10]. With evolving strategies and development of performance indicators in managing appendicitis [13], it is clear we need to review management of this common entity to improve process of delivery of care, outcomes, and reduce hospital re-admissions [14].

This study assessed all patients undergoing appendectomy over a period of one year in a regional university hospital to identify opportunities to improve diagnostic accuracy and outcomes.

## 2. Methods

### 2.1. Study Design

This is an ethically approved retrospective cohort study of all consecutive patients, including both adult and paediatric presentations, undergoing appendicectomy between March 2016 and March 2017 at Letterkenny University Hospital in Ireland. It is a 330-bed regional hospital serving a population of 160,000, with a complement of five consultant surgeons in the surgical department, specialising in emergency and breast, colorectal, upper GI, and three general surgeons. During the study period, 8 consultant surgeons managed both adult and paediatric patients. The emergency roster is 1 week on in 5 and is consultant-led with 1 senior and 1 junior resident on rotating call from a pool of 14 residents. Imaging was always performed and reported by a consultant radiologist.

### 2.2. Patient Demographics

Data including age, gender, day of admission, time from admission to surgery in minutes and length of stay in days was collected. Paediatric patients were defined as aged between 0 and 15 years, and adults were defined as aged 16 years and above. The final radiological report was correlated with the final histology to calculate accuracy of radiological investigations. There was no standardised radiology reporting proforma. The Charlson Comorbidity Index (CCI) was used to classify patients based on severity of pre-existing conditions by assigning grade 0 for no comorbidities, 1 for a single chronic illness and 2 for more than 1 chronic illness [15].

### 2.3. Factors Associated with Operative Data

The preferred surgical approach was laparoscopic with all but one surgeon and conversion rates were recorded. Intraoperative findings were categorised using the American Association for the Surgery of Trauma (AAST) grades, where grade 1 was assigned to acutely inflamed appendix, grade 2 to gangrenous appendix, grade 3 to perforated appendix with local contamination, grade 4 to perforated appendix with peri appendiceal phlegmon or abscess and grade 5 to perforated appendix with generalised peritonitis [16]. Intraoperative reported findings of acute appendicitis had to be confirmed histologically before being deemed as true acute appendicitis. Negative appendicectomy (NA) was defined as histologically normal appendix. Enterobious vermicularis infestation in the absence of inflammation was classed as a normal appendix. Complications were recorded using the Clavien–Dindo grading system [17]. Patients admitted with right iliac fossa pain who did not have appendectomies were not included in the study.

### 2.4. Statistical Analysis

Data was expressed as mean and standard deviation for normally distributed data and medians and interquartile range for non-normal data. The area under the receiver operating curve (AUC) at the optimal cut-off threshold scores was calculated for the Alvarado score, Appendicitis Inflammatory Response (AIR) score, and Adult Appendicitis Score (AAS) [3,7,8,9,10]. The independent chi-square test was used to determine the relationship between the grade of appendicitis and the negative appendectomy rate (NAR) amongst individual consultants. The consultants were divided into two groups based on a NAR greater, less than and equal to 25%. A *p*-value of less than 0.05 was deemed statistically significant.

### 2.5. Results

A total of 201 patients were studied, 115 male and 86 females, of which 136/201 (67.6%) were adults and 65/201 (32.3%) paediatric. Of the adult group, 83 were male and 53 were female, and of the paediatric group, 32 were male and 33 were female. Median age was 20 years (range: 5 years to 81 years) and no patient below the age of 5 years had an appendectomy during our study period. All patients were admitted via the emergency department and median time from triage to surgical review was 2 h and 38 min, (range: 10 min to 26 h and 10 min). Median time from emergency department review to surgical review was 55 min (range: 5 min to 6 h and 43 min). Median time to operating theatre was 21 h from admission (range: 45 min to 140 h and 30 min). Out of the total, 173 patients (86.1%) underwent laparoscopic approach, 28 (13.9%) had an open approach and 12 (6.9%) of the 173 were converted to open. Acute appendicitis occurred in 166/201 (82.6%). The grade of appendicitis was 1 in 97 (58.4%), grade 2 in 17 (10.2%), grade 3 in 13 (7.8%), grade 4 in 10 (6%) and grade 5 in 29 (17.5%). The negative appendicectomy rate was 35/201 (17.4%) with the individual surgical consultant rates ranging from 6.5–38.9%. (Table 1) The independent chi-square test was performed to examine the relationship between the NAR of greater or less than or equal to 25% among individual consultants and the grade of appendicitis showed no significant association between the two parameters, X^2^ (df4, *N* = 166) = 8.8, *p* = 0.07 (Table 2).

Imaging was performed in 118/201 (58.7%). Abdominal ultrasound (US) was undertaken in 53 (26.4%), of which 39 (73.6%) were female and 14 (26.4%) male. Abdominal CT was done in 59 (29.2%), with 33 (55.9%) male and 26 (44.1) female, and both US and CT in 6 (3%), with 3 (50%) male and 3 (50%) female. US correctly identified acute appendicitis in 14 patients (true positives) and failed to identify appendicitis in 20 patients (false negatives). US reported 16 patients correctly with a normal appendix (true negative), and incorrectly diagnosed 3 patients with appendicitis (false positives). US had a sensitivity of 41.2%, specificity of 84.2%, positive predictive value of 82.4%, negative predictive value of 44.4%, and an accuracy of 56.6%. CT correctly diagnosed acute appendicitis in 55 patients (true positives) and erroneously diagnosed it in 2 (false positives). One patient was correctly reported as normal appendix (true negatives) and one was inaccurately reported as normal (false negative). CT had a sensitivity of 98.2%, a specificity of 88.9%, positive predictive value of 96.5%, negative predictive value of 94.1% and an accuracy of 96%. In patients who had both, the US scan had missed acute appendicitis in 4 patients, and they were correctly diagnosed on CT. CT confirmed the normal appendix in 2 patients as their US was inconclusive, or normal. (Table 3).

**Figure 1:**
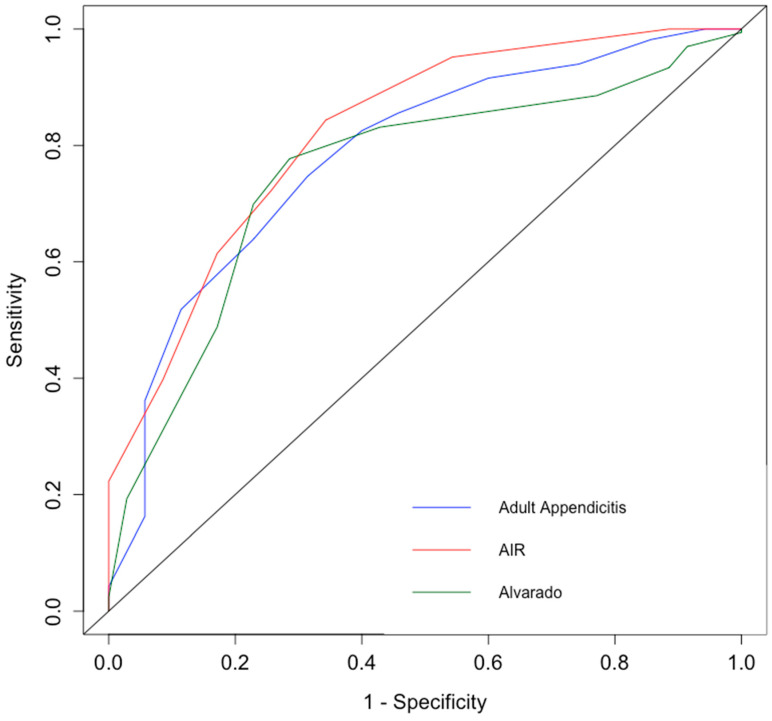
Combined receiver operating characteristic (ROC) curves for the AIR, AAS and Alvarado scoring systems.

The AUC for AIR score was 82.3%, 78.3% for AAS and 75.2% for Alvarado score (Figure 1). The best cut-off point was 4 for AIR, 9 for AAS, and 7 for Alvarado score. If the best cut-off was considered as an interpretation benchmark for these three scoring systems, AIR achieved sensitivity of 84.3% and specificity of 65.7%; AAS would have sensitivity of 74.7% and specificity of 68.6%; while sensitivity and specificity of the Alvarado score would be 77.7% and 71.4% respectively.

Twenty-four (11.9%) were re-admitted, due to pain in 16 (58.3%), collections in 3 (25%), 1 (4.2%) wound abscess, 1 (4.2%) stump appendicitis, 1 (4.2%) small bowel obstruction and 1 (4.2%) fresh rectal bleeding. CT guided drainage was performed in 2 (8.3%). One patient had release of wound collection under general anaesthetic, whereas another patient had laparoscopic drain placement. A laparotomy was undertaken in 3 (12.5%) patients with division of adhesions in 1, the appendicular stump removed in 1 and 1 had multiple collections drained.

## 3. Discussion

This study at a medium-volume university hospital with a mixed general surgical practice, managing emergency surgical needs of both adult and paediatric populations through consistent consultant and trainee staff over a one-year period identified significant variation in outcomes. Appendicitis in this study population was predominantly an adult disease, where no infant, below five years of age, had an appendectomy. This is in keeping with other studies, suggesting that appendicitis is currently increasing in older age groups and it is no longer just in the realm of paediatric surgeons [18,19,20]. It reflects the increasing complexity of adult emergency general surgery decision making and the need for quality and performance [13,21]. Having guidelines, while commendable, needs to be supplemented by robust systems to ensure surgery, when performed, is actually required [7,10]. 

There has been debate about whether paediatric appendicitis and its management is a separate entity from adult appendicitis. It may pose a diagnostic dilemma, especially in non-verbal infants and younger children. Others have concluded that there may be no significant difference in diagnosis and management of children compared to adults [22,23,24]. Bansal et al. suggested that the very young patients present with more advanced appendicitis but are less likely to develop post-operative abscesses [24]. Peculiarly, patients below the age of five were not operated on in our cohort, and Pogorelic and colleagues, in their review of appendicitis, found that children under the age of 5 years account for less than 5.4% of the paediatric population [23]. Furthermore, with regards to diagnostic imaging and management, recent guidelines have suggested ultrasound should be the choice of initial imaging, and although previously debated, the use of laparoscopic approach tends to be favourable even for children [10]. We found that 81.5% (53/65) of all paediatric patients in our study were operated on via laparoscopic approach, which was also very similar to our adult cohort.

A laparoscopic approach was undertaken in 86.6%. This is slightly lower than the international average and reflects one surgeon’s practice who maintains an open approach [21].

The acute appendicitis rate of 82.6% is consistent with the international literature, but recent studies suggest a slightly higher rate of negative appendectomies in the UK than in this current study [5,7,25]. A NAR of 17.4% is unacceptable. In adults this was 14.7% (20/136); 10.8% (9/83) in males and 20.8% (11/53) in females. The paediatric NAR was 23.1% (15/65); 12.5% (4/32) in boys and 33.3% (11/33) in girls. Our study is in keeping with the literature which shows female patients have higher negative appendectomy rates and morbidity associated with it [21,26,27,28]. Mackey et al. recently identified factors that may be associated with higher negative appendicectomies, including independent factors such as age below 27, duration of symptoms for more than 48 h, normal leucocyte count, use of single imaging modality (ultrasound or CT) and time to operation greater than 24 h. Combination of factors like symptoms more than 48 h, normal leucocyte count, and a macroscopically normal intraoperative appendix may also be associated with a negative appendicectomy, but they also discussed that an intraoperatively normal looking appendix was histologically confirmed as normal only in 41%, hence the decision to not perform an appendicectomy in the absence of other pathologies still requires further studies where long-term effects of the practice are highlighted [29].

The incidence of appendicitis globally is variable, but averages at about 100–112 per 100,000 person-years in the United States and Europe, respectively [5,19]. The literature also shows that appendicitis is more prevalent in males than females [18,27,30]. Despite being one of the most common presentations requiring acute surgical intervention, the diagnosis and surgical management of acute appendicitis remains challenging. Female patients may have multiple causes for presenting with lower abdominal pain and further investigation with ultrasound may be beneficial, but the risk of radiation exposure of CT scans leads the surgeons to choose diagnostic laparoscopy as an option, and hence, higher incidence of negative appendicectomies, as the literature has shown that intra operatively ‘normal’ looking appendix may still have histological evidence of inflammation in up to 29% of cases [26,31,32]. Therefore surgeons tend to perform appendectomies during diagnostic laparoscopies if no other explanation of pain is evident [26,33,34]. Furthermore, the range of negative appendectomy varied between surgeons in our study, which further indicates and confirms the complexity and variability that occurs in surgical decision making. The explanation of surgical variance and why three surgeons had a NAR of above 25% is challenging and probably multifactorial. The figures could be confounded by the concept of utilising a diagnostic laparoscopy and then removing a normal appendix, but this was not the intent in the cases in our study. Surgical decision making and assimilation of information from multiple sources, both clinical and laboratory, make the surgical decision open to interpretation and human error. It begs the question about the introduction of mandatory outcome measures. Sherratt et al., in their recent study, incorporating 147 stakeholders, both adult and paediatric surgeons, patients and their families, identified re-operation and recurrent intraabdominal abscesses as the most important negative outcomes, but also included NAR [35].

Our study identified that time to undertake patient review by the emergency department staff and surgical team was prompt. The median waiting time to theatre was 21 h from admission, which was slightly longer than Foley et al., who had a median waiting time of 18 h [36]. A recent meta-analysis by van Dijk et al. concluded that a waiting time of greater than 24 h may be acceptable for patients who do not show signs of complicated appendicitis at the time of admission [37]. Other studies have deemed 18 h as an upper limit for safe inpatient waiting times [38,39]. A new pathway with easier access to the emergency operating theatre needs to occur. Mackay et al. suggested that a delay in surgery beyond 48 h may be a predictor of negative appendicectomy, as the urgently operated patients usually have a clear presentation, whereas patients where diagnostic laparoscopy is the final investigation, an appendicectomy may be done contributing to morbidity associated with surgery. Instead, they suggest that the role of re-imaging needs consideration as a cost-effective and less invasive option [29].

The use of imaging is variable where a CT scan is almost universally performed in the United States as opposed to rarely performed in the UK [6]. In this study, it reflected the ‘in between’ picture with imaging performed in over half of the group. Ultrasound was found to have a sensitivity of only 36.8% and an overall accuracy of 54%; this is in keeping with literature as recent studies have shown a similar sensitivity of 36% [7,40]. However, point of care ultrasound may be a useful and cost-effective tool to aid in the diagnostic pathway [10]. CT scan, on the other hand, is highly accurate in diagnosing appendicitis but may have a significant impact on patients’ long-term outcomes due to the exposure to radiation leading to risk of cancer, which is higher in the younger population [41,42]. To understand the impact of the ionising radiation dose, Smith-Birdman et al. explained that the long- term survivors of the Hiroshima and Nagasaki atomic bombs had a radiation exposure of 10–100 milliSieverts (mSv), and they had a higher risk for developing cancer [42]. The effective radiation dose received from a single CT abdomen and pelvis is highly variable between countries in Europe ranging from 7.3 mSv in Switzerland to 15.7 mSv in Israel. This, coupled with the fact that younger patients would have multiple scans throughout their life span, leads to significant increase in the risk of developing cancer. Therefore, a balance is suggested in the Jerusalem Guidelines, where US scanning may still help reduce CT scanning by 50% and also, backed by the OPTICAP study, utilisation of low-dose CT scanning may reduce the mean radiation dose to 3.33 mSv while maintaining the accuracy [10,43,44]. In our study, the combined conditional approach to CT was used in only 3%, whereas a combined approach may reduce negative appendicectomy rate as well, and may be considered as the approach of choice in the future [29].

Appendicitis scoring systems were not routinely undertaken or documented in this study, but were all retrospectively performed, where the AIR score had the highest sensitivity but the lowest specificity. Bhangu and the Right Iliac Fossa Treatment (RIFT) study group attempted to validate 15 risk prediction models for patients with suspected acute appendicitis. AAS proved to have a high specificity for the female cohort with a false positive rate of less than 5%, and the AIR score was the optimal model for men with a false positive rate of 2.4%. They recommended the use of Shera’s score for the paediatric patients [7]. It was pointed out by Anderson that it is recommended to repeat the scoring system during observation of patients with persistent symptoms and biochemical markers to come to a diagnostic decision [45]. The recent guidelines also suggest that the AIR and the AAS significantly aid in clinical diagnosis and have a potential for reducing negative appendectomy rates as well as limiting the use of imaging [10,46,47,48,49].

Unplanned 30-day re-admissions occurred in 11.4% and this was higher than the international average, as a recent meta-analysis by Bailey et al. identified an average re-admission rate of 4.5% as a benchmark [14,50,51]. The factors associated with a higher risk of re-admission are the presence of diabetes, an open surgical approach and complicated appendicitis [14,52]. Anticipating these risks and tailoring the approach to patients’ clinical course could further help in reducing the re-admission rates. Furthermore, a robust system of data capturing that which identifies the cause of re-admission may help us focus on key elements for improvement. This study identified that the leading cause of re-admission was suboptimal pain management and intra-abdominal collection, which is similar to the results of Moghadamyeghaneh et al., who reported intra-abdominal infection and pain as the leading causes of re-admissions [53].

Limitations of the study include that it is a retrospective review and is institutional specific. Patient preferences and their satisfaction were not analysed in our study and including them in prospective analysis may help formulate better management strategies [54,55]. Long term follow-up was not undertaken.

## 4. Conclusions

This paper identified the opportunity to improve diagnosis of acute appendicitis and potentially reduce variance among surgeons. This could be aided by decision tree pathways combined with the mandatory use of scoring systems which have been shown to reduce negative appendectomy rate. Ultrasound provided little benefit and its value outside the female patients needs to be questioned. A re-admission prevention strategy needs to be implemented to reduce the rate of re-admission. The paper challenges concepts in surgeons’ clinical responsibility, education and governance, which are all crucial in improving outcomes. 

## Figures and Tables

**Table 1:** Outcomes for individual consultants.

Consultant	Total Appendectomies	Laparoscopic	Open	Conversion to Open (%)	Negative Appendectomy	(NAR%) (Negative Appendectomy Rate)
1	49	38	5	6 (13.6)	4	8.2
2	36	32	1	3 (8.3)	14	38.9
3	31	30	1	0	2	6.5
4	27	26	1	0	4	14.8
5	19	17	1	1 (5.6)	5	26.3
6	15	8	5	2 (20)	2	13.3
7	13	0	13	0	1	7.7
8	11	10	1	0	3	27.3
**Total**	**201**	**161**	**28**	**12 (6.9)**	**35**	**17.4**

**Table 2:** Negative appendectomy rate (NAR) 25% and appendicitis grade (AAST) of consultants.

**(NAR) > 25%**								
**Consultant**	**NAR (%)**	**Total**	**Normal**	**Grade 1**	**Grade 2**	**Grade 3**	**Grade 4**	**Grade 5**
2	38.9	36	14	17	1	0	0	4
5	26.3	19	5	9	0	2	1	2
8	27.3	11	3	7	0	0	0	1
**Total**		**66**	**22**	**33**	**1**	**2**	**1**	**7**
**(NAR) ≤ 25%**								
**Consultant**	**NAR (%)**	**Total**	**Normal**	**Grade 1**	**Grade 2**	**Grade 3**	**Grade 4**	**Grade 5**
1	8.2	49	4	24	8	2	5	6
3	6.5	31	2	12	5	1	2	9
4	14.8	27	4	14	2	4	0	3
6	13.3	15	2	9	0	0	2	2
7	7.7	13	1	5	1	4	0	2
**Total**		**135**	**13**	**64**	**16**	**11**	**9**	**22**

**Table 3:** Diagnostic performance of imaging modalities.

	Patients Imaged (%) *	Male (%)	Female (%)	Sensitivity (%)	Specificity (%)	Accuracy (%)	Positive Predictive Value (%)	Negative Predictive Value (%)
**US**	53 (26.4)	14 (26.4)	39 (73.6)	41.2	84.2	56.6	82.4	44.4
**CT**	59 (29.4)	33 (55.9)	26 (44.1)	98.2	88.9	96	96.5	94.1

* Total number of patients was 118/201 (58.7%) where 6 (3%) patients had both ultrasound and CT scan.
